# *Enterobius vermicularis* causing acute appendicitis, a case report with literature review

**DOI:** 10.1016/j.ijscr.2019.09.025

**Published:** 2019-09-25

**Authors:** Zuhair D. Hammood, Abdulwahid M. Salih, Shvan H. Mohammed, Fahmi H. Kakamad, Karzan M. salih, Diyar A. Omar, Marwan N. Hassan, Shadi H. Sidiq, Mohammed Q. Mustafa, Imad J. Habibullah, Drood C. Usf, Anmar E. Al obaidi

**Affiliations:** a Sulaimani Teaching Hospital, Sulaimani, Kurdistan, Iraq; b Faculty of Medical Sciences, School of Medicine, Department Surgery, University of Sulaimani, Sulaimani, Kurdistan, Iraq; c Kscien Organization, Hamdi Str., Azadi Mall, Sulaimani, Kurdistan, Iraq; d Chara Laboratory, Shahedan Street, Kalar, Kurdistan, Iraq; e Faculty of Medical Sciences, School of Medicine, Department Cardiothoracic and Vascular Surgery, University of Sulaimani, Sulaimani, Kurdistan, Iraq; f Erbil Polytechnic University, Shaqlawa Technical Institute, Erbil, Kurdistan, Iraq; g Medical Analysis Department, Science Faculty, Ishik university, Erbil, Kurdistan, Iraq

**Keywords:** Acute, Abdomen, Tenderness, Infestation, Parasite

## Abstract

**Introduction:**

*Enterobius vermicularis* is one of the commonest parasitic infestations worldwide but its association with acute appendicitis remains controversial. It is very rarely encountered during appendectomy. The aim of this paper is to report a case of acute appendicitis caused by *Enterobius vermicularis*. A 23-year-old housewife presented with a right lower abdominal pain for the past 8 h. Clinical examination revealed right iliac fossa tenderness upon palpation and rebound tenderness upon release. The patient was diagnosed as a case of suspected acute appendicitis. The patient was anesthetized and intubated. Delivery of the vermiform appendix done through right grid iron incision. Intra operatively an inflamed appendix obstructed by *Enterobius vermicularis* was noted.

**Conclusion:**

*Enterobius vermicularis* can habit the appendix and induce the signs and symptoms of A.A with or without actual histopathological acute appendicitis. The treatment of choice is surgical resection of the appendix.

## Highlights

• *Enterobius vermicularis* is one of the commonest parasitic infestations worldwide.
• Its association with acute appendicitis remains controversial.
• It is very rarely encountered during appendectomy.
• In this report, a case of acute appendicitis caused by *Enterobius vermicularis* has been presented.

## 1 Introduction

Acute appendicitis (AA) represents one of the commonest causes for emergency operations worldwide with a cumulative lifetime incidence rate of 9.0%, accounting to a significant portion of intraabdominal conditions. During the 20th century, the disease was mostly reported within the western countries, however a rise in its incidence has been noted within newly industrialized countries in 21^st^ century [1]. In the United States only, annually more than 70,000 children are diagnosed with appendicitis during hospital admission [2]. Over the age of 40 years, appendicitis may have more morbid causes, like caecal cancer among other colorectal pathology, though neoplasm is uncommon but should not be ignored in this age group [3]. Parasitic infestation represents one of the controversial etiologies for acute appendicitis and their relation has been in debate [4]. However *Enterobius vermicularis* remains one of the commonest parasitic infestation worldwide, with an estimate of 209 million affected people and often it is referred to as threadworm or pinworm [5]. Although perianal pruritus is the most common manifestation of *Enterobius vermicularis* (pinworms), pinworms have been reported to be found in different multiple locations, including the vermiform appendix. Regarding appendiceal helminthes, recent literature concentrate mainly on the pathological changes that caused by the presence of intraluminal parasites [6]. In this paper we present an adult patient with acute appendicitis caused by *Enterobius vermicularis* and go over the literature briefly. The case has been reported in line with SCARE guideline [7].

### 1.1 Patient information

A 23-year-old housewife patient presented to Emergency Department with a right lower abdominal pain for the past 8 h with concomitant anorexia, nausea and vomiting twice. Other than having a mild fever, the patient had normal vital signs.

### 1.2 Clinical findings

Clinical examination revealed right iliac fossa (RIF) tenderness upon palpation and rebound tenderness upon release. Other signs like Rovsing and pointing signs were positive as well.

### 1.3 Diagnostic assessment

Complete blood count revealed mild leukocytosis (12,000 m/mm^3^) while other routine investigations like urinalysis, blood urea and serum creatinine were not remarkable. Abdominal and pelvic ultrasound reported no unusual findings and pregnancy was excluded through blood test. The patient was diagnosed as a case of suspected acute appendicitis (S.A.A.).

### 1.4 Therapeutic intervention

**Fig. 1:**
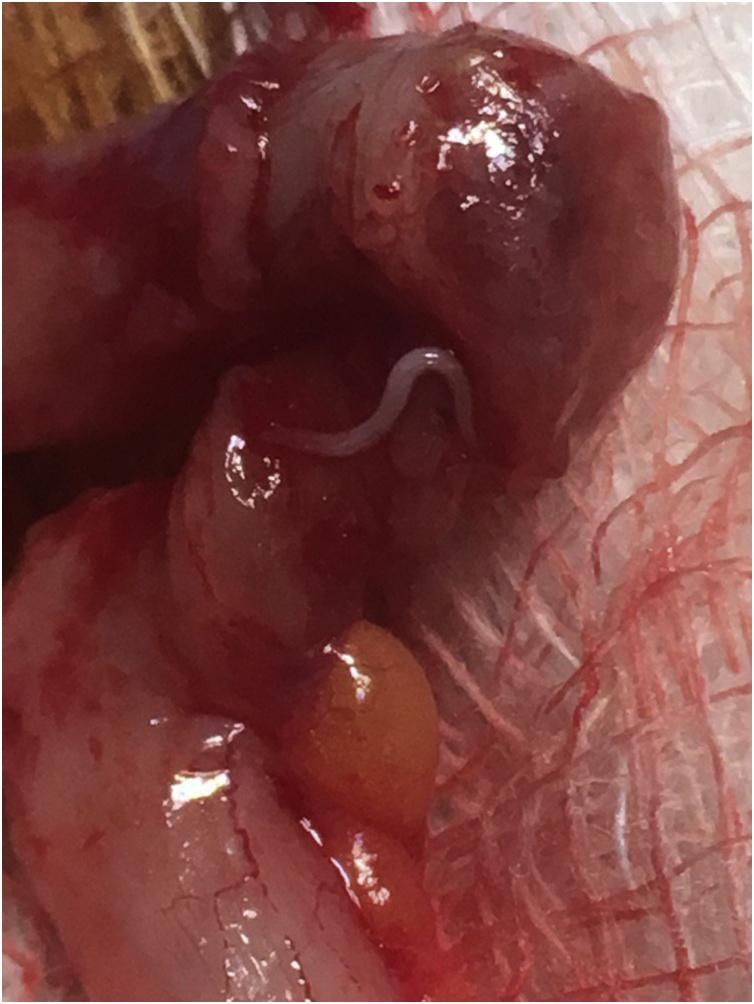
Intraoperative picture showing *Enterobius vermicularis* through the wall of the appendix.

**Fig. 2:**
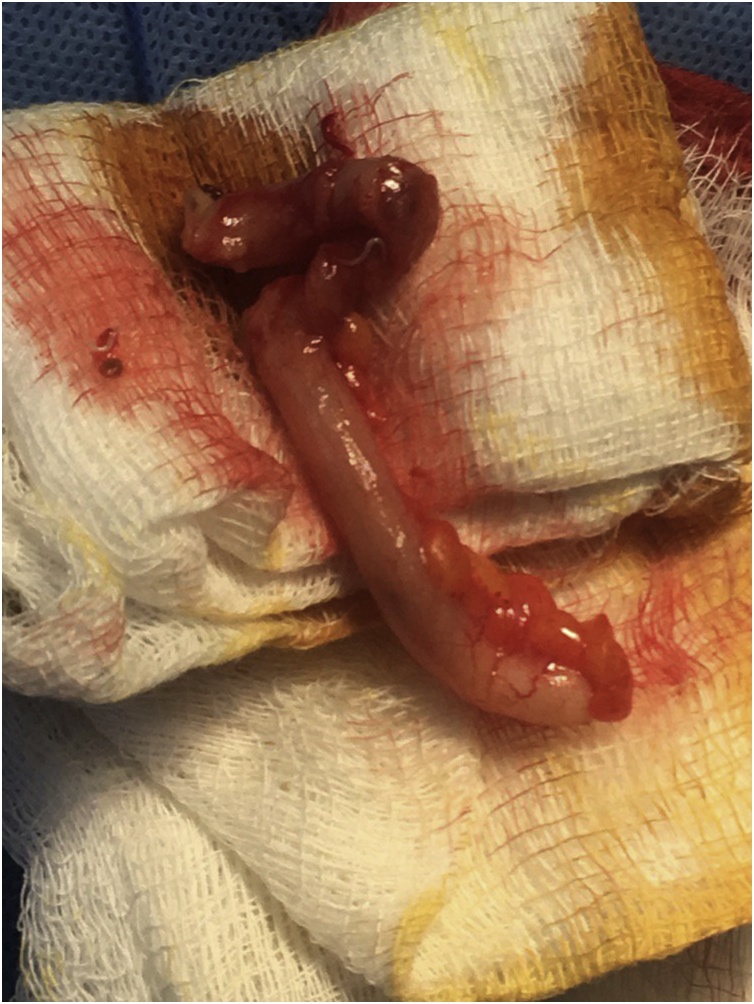
Appendix after resection with the word seen inside the lumen.

The condition was explained to the patient and informed consent was taken for surgery. During hospital stay she received 1 unit of normal saline (500 cc) and then was taken into the operation theatre. Pre-operative prophylactic antibiotic given (single dose of Ceftriaxone vial 1 g). The patient was anesthetized with General Anesthesia (GA) and intubated. Delivery of the vermiform appendix done through right grid iron incision. Intra operatively an inflamed appendix obstructed by *Enterobius vermicularis* was noted (Fig. 1, Fig. 2). An eventful classical appendicectomy done. Terminal ileum and right ovary checked and both were normal.

### 1.5 Follow-up and outcomes

The patient recovered without any complications and was transferred to the surgical ward for observation. Within 8 h, she passed flatus and started oral feeding. After 24 h, she was sent home in a good heath condition and scheduled to visit after 8 days, where she was healthy and the would stiches removed.

## 2 Discussion

*Enterobius vermicularis* is known by many names (seatworm, pinworm, oxyuriasis, threadworm) and first description of human infestation nearly dates back 10,000 years. However, it was Fabrius in 1634 who first described involvement of the worm in appendicitis. Once *E. vermicularis* reaches maturity, it stays and reproduces in terminal ileum, caecum, appendix and ascending colon. The lifecycle of the male worm ends after fertilization and dies, while the female must migrate to the anal canal to lay eggs [8]. *The lifespan of Enterobius vermicularis (pinworm) is between 2 and 5 weeks(panidis).* Despite that the relationship between *E. vermicularis* and pathogenesis of appendicitis had been studied for many years, the influence of the parasite to induce inflammation is still unclear. Although *E. vermicularis(pinworm)* may have a role in causing appendiceal disconfort or appendiceal chronic inflammation due to obstruction, the majority of cases have no acute inflammation [8]. The belief that Enterobius infestation can cause diseases like acute appendicitis, chronic appendicitis and ruptured appendicitis is shared by others [6], and even more morbid complications like gangrenous appendicitis and perforation resulting in peritonitis [9]. None the less, there have been reports of completely asymptomatic patients [6]. The “appendiceal syndrome” represents clinical symptoms without acute inflammation and is also referred to as appendiceal colic, where the patient has an intermittent chronic pain in right lower quadrant and pelvic region, and appendiceal obstruction presents a reasonable hypothesis for the condition [9]. Once the worm is detected in theatre, the patient should be treated for systemic infestation in case there are subclinical infestation elsewhere. The diagnosis of AA is mostly clinical and proper history, physical examination and a raised inflammatory parameter help with the diagnosis [10]. To this day, the gold standard treatment for AA is appendectomy, and interval appendectomy nowadays is not uncommon [11]. Non-Operative Management (NOM) of AA has been proposed by several randomized controlled trails 2–8 and meta-analyses 7,9–15 and see it as viable option along with the century standing practice of surgical resection [12]. Appendectomy in these patients are merely a treatment for a complication, but the root cause is still there. Pyrantel pamoate is the drug of choice for *Enterobius vermicularis* treatment. It is an agent that blocks neuromuscular depolarization making the worm undergo spastic paralysis through continuous nicotinic activation, ultimately the worm detaches from the host and consequently will be expelled through defecation [13]. Table 1 reviews the most important reports regarding acute appendicitis and *Enterobius vermicularis*.

**Table 1:** The most prominent reports describing acute appendicitis with *Enterobius vermicularis*.

Authors/references	No. of cases positive for E. V/year of publication	Country of report	Gender distribution/ mean age	Inflamed appendix
Akkapulu [14]	9/2015	Turkey	7 females, 2 males/ 31	One case
Balci [15]	1/2018	Turkey	Female/ 35 years	One case
Budd [16]	38/1987	UK	Not found	14 cases
Chilkar [17]	1/2016	India	Male/ 8 years	Suppuration
Cruz [18]	1/2012	Brazil	Female/29 years	One case
Dahlstroam [19]	63/1994	Australia	--/22.8 years	23 cases
Dunphy [20]	1/2017	UK	Female/10 years	None
Efared [21]	1/2017	Morocco	Male/ 21 years	One case
Efraimidou [22]	1/2008	Greece	Female/ 15 years	None
Eleftherios [8]	7/2012	Greece	4 females, 3 males/25 years	None
Fleming [23]	13/2015	Ireland	--/11.4 years	4 cases
Habashi [24]	1/2019	Canada	Male/ 9 years	One case
Hamdona [25]	30/2013	Palestine	17 male/ 13 female/---	23 cases
Harris [26]	22/1925	USA	19 females 3 males/ 23 years	---
Lala [27]	109/2014	New Zealand	--/11.6 years	27 cases
*Vleeschouwers* [28]	1/2013	Belgium	Female/ 17 years	None
Madhukar [9]	1/2014	India	Female/ 18 years	one
Maki [29]	16/2012	USA	--/9.5 years	Two cases
Panidis [5]	1/2011	Greece	Female/52 years	None
Ramezani [30]	144/	Iran	82 female, 62 male/20.4 years	76 cases
Risio [31]	1/2016	Iran	Female/23	One case
Sah [32]	9/ 2006	Nepal	--/15 years	3 cases
Upadhyaya [33]	6/ 2015	Nepal	---/--	None

In composite, *Enterobius vermicularis* can habit the appendix and induce the signs and symptoms of A.A with or without actual histopathological acute appendicitis. The treatment of choice is surgical resection of the appendix.

## Sources of funding

No source to be stated.

## Ethical approval

Approval has been taken from Kscien centre.

## Consent

Consent has been taken from the patient and the family of the patient.

## Author contribution

Zuhair D. Hammood and Abdulwahid M. Salih: Surgeon performed the operation and follow up.

Shvan H. Mohammed, Fahmi H. Kakamad, and Karzan M. salih: Writing the manuscript and follow up.

Diyar A. Omar, Marwan N. Hassan, Shadi H. Sidiq, Mohammed Q. Mustafa, Imad J. Habibullah, and Drood C. Usf: literature review, final approval of the manuscript.

## Registration of research studies

Not applicable

## Guarantor

Fahmi Hussein kakamad.

## Provenance and peer review

Not commissioned, externally peer-reviewed.

## Declaration of Competing Interest

There is no conflict to be declared.
